# Revealing the pathogenic and ageing-related mechanisms of the enigmatic idiopathic pulmonary fibrosis (and chronic obstructive pulmonary disease)

**DOI:** 10.1097/MCP.0000000000000876

**Published:** 2022-06-24

**Authors:** Paolo Spagnolo, Umberto Semenzato

**Affiliations:** Respiratory Disease Unit, Department of Cardiac Thoracic, Vascular Sciences and Public Health, University of Padova, Padova, Italy

**Keywords:** ageing, autophagy, chronic obstructive pulmonary disease, Idiopathic pulmonary fibrosis, mitochondrial dysfunction, senescence

## Abstract

**Purpose of review:**

Growing evidence suggests that ageing-associated alterations occur in both idiopathic pulmonary fibrosis (IPF) and chronic obstructive pulmonary disease (COPD). Here, we review the most recent literature on dysregulated ageing pathways in IPF and COPD and discuss how they may contribute to disease pathogenesis.

**Recent findings:**

Recent studies have shown that alveolar epithelial type II (ATII) cells undergo premature senescence under stress and that senescent ATII cells promote lung fibrogenesis. Some studies have explored the role of mitochondrial dysfunction in IPF. They have provided evidence that dysfunctional mitochondria are important contributors to fibrogenesis through release of damaged DNA and excessive formation of reactive oxygen species, whereas restoration of mitochondrial homeostasis may attenuate lung fibrosis. Insufficient autophagy has been shown to promote epithelial-to-mesenchymal transition and aberrant epithelial-fibroblast crosstalk, suggesting that autophagy augmentation may represent a potential therapeutic strategy. A number of studies have also explored the role of cellular senescence, mitochondrial homeostasis and autophagy in COPD.

**Summary:**

Several ageing mechanisms are dysregulated in the lungs of patients with IPF and COPD, although how they contribute to disease development and progression remains elusive. Genetic or pharmacologic attenuation of senescence-related pathways and elimination of senescent cells may represent a promising therapeutic strategy.

## INTRODUCTION

Idiopathic pulmonary fibrosis (IPF) is a chronic, progressive, smoking- and age-related interstitial lung disease of unknown origin with a poor prognosis and limited therapeutic options [1]. The mechanisms involved in disease pathogenesis are poorly understood, but they are likely to involve host (genetic and epigenetic) and environmental factors, leading to aberrant activation of alveolar epithelial cells (AECs). Dysfunctional AECs release a plethora of profibrotic mediators that induce the expansion of subsets of aggressive and apoptosis-resistant fibroblasts/myofibroblasts that secrete excessive extracellular matrix (ECM) material leading to distortion of the lung architecture and irreversible organ failure [2^▪^]. Chronic obstructive pulmonary disease (COPD) is also a smoking- and age-related disease, although the pathological features of IPF and COPD differ markedly, with the former being characterized by progressive scarring of the lung parenchyma and reduced lung volumes, and the latter by destruction of the lung interstitium leading to formation of emphysematous spaces and increased lung volumes [3]. IPF and COPD differ greatly also with regard to prevalence; in fact, while about 10 in 10 000 individuals are diagnosed with IPF after the age of 75 [1], around 1200 in 10 000 are diagnosed with COPD after the age of 65 years [4]. 

**Box 1:**
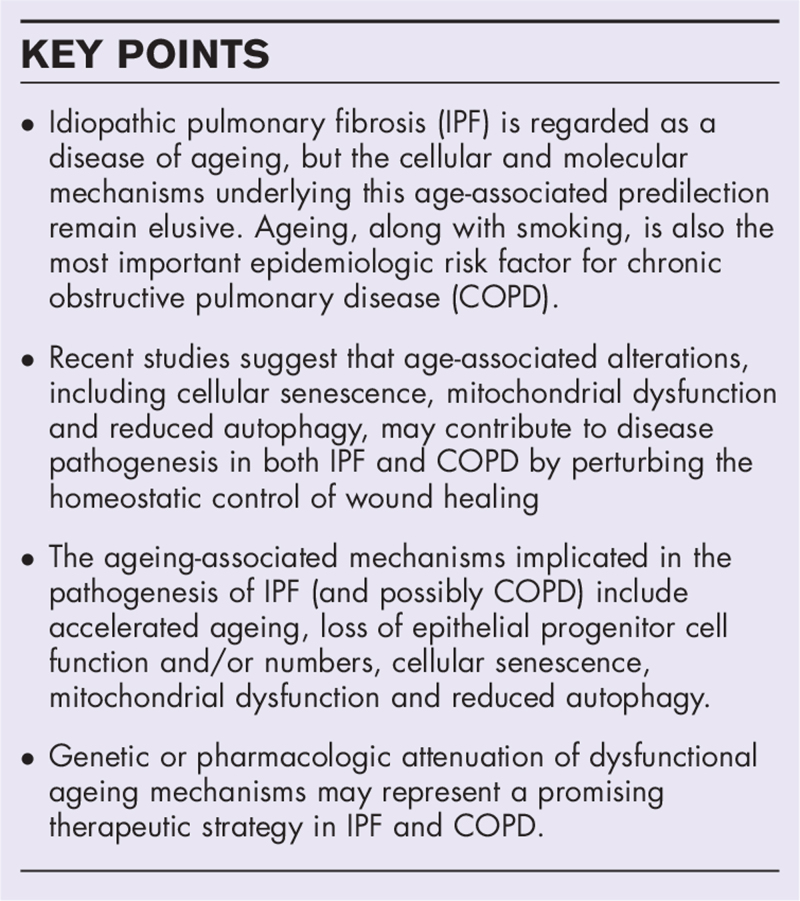
no caption available

Although ageing represents the most important epidemiologic risk factor for IPF, the reasons for its age-associated predilection are incompletely understood. Lung fibrosis results from an abnormal reparative process that is unable to restore organ structure following injury. A number of ageing mechanisms, including senescence, mitochondrial dysfunction, and diminished autophagy, are believed to contribute to perturbed homeostatic control of wound healing, thus increasing susceptibility to the disease [5]. However, even in normal lung tissue, ageing is associated with increasing expression of genes involved in cellular senescence, progressive loss of epithelial cells and increasing proportion of fibroblasts, consistent with a pro-fibrotic profile [6].

The last few years have witnessed an exponential increase in the number of studies investigating the role of ageing in IPF. In this article, we summarize some of such studies and discuss how dysregulated senescence pathways may contribute to disease pathogenesis in IPF (and COPD).

## CELLULAR SENESCENCE

### Idiopathic pulmonary fibrosis

Cellular senescence is a state of irreversible cell growth arrest that can be triggered by several factors, including ageing, DNA damage, oncogene activation and oxidative stress. Senescence of alveolar epithelial type II (ATII) cells is a pathological feature of IPF, although how senescent ATII cells contribute to lung fibrogenesis remains poorly understood. Rana *et al.*[7] have shown that transforming growth factor (TGF)-β1, one of the most potent profibrotic cytokines, induces plasminogen activator inhibitor-1 (PAI-1), a mediator of cell senescence and fibrosis, and p16, a cyclin-dependent kinase inhibitor and biomarker of ageing, which renders the cell growth arrest irreversible, in primary mouse ATII cells. Notably, deletion or inhibition of PAI-1 activity blocks TGF-β1-induced senescence as well as a senescence-associated secretory phenotype (SASP) in ATII cells whereas silencing of p16 ameliorates PAI-1-induced ATII cell senescence. Yao and colleagues used loss of Sin3a – a transcription regulator – in adult mouse ATII cells to initiate a program of p53-dependent cellular senescence, ATII cell depletion, and spontaneous pulmonary fibrosis [8^▪^]. Interestingly, they showed that senescence rather than loss of ATII cells promotes progressive fibrosis, thus suggesting that genetic or pharmacologic attenuation of senescence-related pathways and elimination of senescent cells may represent a potential therapeutic approach.

ATII stem cells undergo premature senescence under stress, but the associated mechanisms remain unclear. Wang *et al.*[9^▪▪^] have recently shown that in response to radiation, oxidative stress, or bleomycin, the E3 ubiquitin ligase FBW7 binds to telomere protection protein 1 (TPP1), thus triggering telomere uncapping and DNA damage response, and leading to cell senescence and tissue fibrosis. Inhibition of FBW7 or overexpression of TPP1 reduces telomere uncapping and shortening, and expands the ATII stem cell population in mice, thus elucidating a pivotal mechanism underlying stress-induced ATII stem cell senescence and fibrosis.

Evidence is accumulating that GMP-AMP synthase (cGAS) is crucial in perpetuating senescence by binding damaged DNA released into the cytosol. Recently, Schuliga *et al.*[10] examined the contribution of cGAS and self-DNA to the senescence of lung fibroblasts in IPF. They observed cGAS immunoreactivity in fibrotic regions associated with fibroblasts in lung tissue of IPF patients, and provided evidence that cGAS reinforces lung fibroblast senescence involving damaged self-DNA, thus suggesting that targeting of cGAS to suppress senescent-like responses may have potential therapeutic implications in IPF. Endoplasmic reticulum (ER) stress has also been suggested as a potential mechanism linking ageing to IPF. Borok *et al.*[11^▪^] evaluated the role of ER stress in AEC dysfunction and fibrosis by studying mice with tamoxifen (Tmx)-inducible deletion of ER chaperone Grp78, a key regulator of ER homeostasis, ATII cells, and IPF lung slice cultures. Grp78 deletion induced a number of features typical of IPF, including spatially heterogeneous fibrosis characterized by *fibroblastic foci* and ATII cell hyperplasia, particularly in old and male mice. Moreover, Grp78 knockout ATII cells showed evidence of ER stress, apoptosis, senescence, and activation of TGF-β/SMAD signalling, supporting a causal role for ER stress and resulting epithelial dysfunction in IPF.

Fibrotic lesions in both IPF lung and experimental pulmonary fibrosis in aged mice highly express mouse double minute 4 homolog (MDM4), a matrix stiffness-regulated endogenous inhibitor of p53 [12]. Reducing matrix stiffness down-regulates MDM4 expression, resulting in p53 activation in primary lung myofibroblasts isolated from IPF patients. In turn, gain of p53 function activates a gene program that sensitizes lung myofibroblasts to apoptosis and promotes the clearance of apoptotic myofibroblasts by macrophages, suggesting that MDM4 may represent a molecular target in ageing-associated lung fibrosis. Overexpression of Sirtuin-3 (SIRT3), a mitochondrial deacetylase downregulated in IPF lungs and in mice following lung injury, has also be shown to restore apoptosis susceptibility in fibroblasts [13]. Myofibroblasts have long been thought of as terminally differentiated cell types [14], but more recent data suggest they retain the capability for dedifferentiation and subsequent proliferation [15]. Kato *et al.*[16] have shown that senescent/IPF myofibroblasts fail to undergo dedifferentiation and become resistant to apoptosis. In addition, senescent/IPF myofibroblasts overexpress the transcription factor MyoD, which acts as a critical switch in the differentiation and dedifferentiation of myofibroblasts, suggesting that MyoD silencing may represent a potential therapeutic strategy in IPF and other fibrotic diseases.

### CHRONIC OBSTRUCTIVE PULMONARY DISEASE

Several cellular senescence mechanisms have been described in COPD. Cigarette smoke (CS) exposure may lead to DNA double-strand breaks and genomic instability, and induce aberrant cytokine secretion with characteristics of SASP [17]. Altered antioxidant mechanisms have also been reported in COPD, including reduced expression of superoxide dismutase and lower blood concentrations of the reduced form of glutathione (GSH) and of members of the GSH-S transferase superfamily [18]. Nuclear factor erythroid-derived 2, like 2 (NFE2L2/Nrf2) is a transcription factor involved in the regulation of the antioxidant response. Nrf2 expression is reduced in macrophages and epithelial cells from COPD patients [19,20] and CS-exposed Nrf2-deficient mice have increased lung inflammation and emphysematous changes [21].

CS exposure increases the levels of markers of prosenescence (i.e., p16, p21, p53) and DNA damage (i.e., γH2AX phosphorylation) in human lung epithelial cells and fibroblasts *in vitro*, acute/chronic CS-exposed mouse lungs *in vivo*, and in lungs of patients with COPD [22–26]. p16INK4a (a cyclin-dependent kinase inhibitor) is associated with cellular senescence *in vitro* and *in vivo*, and removal of p16 delays cellular senescence in premature ageing mice [27,28]. Moreover, CS-exposed p16^−/−^ mice exhibit normal pulmonary function, reduced emphysema, and increased insulin-like growth factor 1 (IGF1) signalling, suggesting that targeting senescence may facilitate alveolar regeneration in COPD [29]. However, genetic ablation of p16 does not prevent cellular senescence and airspace enlargement in a mouse model of COPD, suggesting that p16 is not the only determinant of CS-induced cellular senescence [30]. Recently, Woldhuis *et al.*[31^▪^] have shown that lung fibroblasts from COPD patients secrete higher levels of a number of SASP proteins (many of which have been implicated in chronic inflammation) compared with matched controls, suggesting that senescent fibroblasts may lead to extracellular membrane dysregulation and aberrant tissue remodelling.

## MITOCHONDRIAL DYSFUNCTION

### Idiopathic pulmonary fibrosis

Mitochondria are organelles essential for energy generation, maintenance of cellular metabolism, intracellular signalling and regulation of cell death programs. Growing evidence suggests that mitochondrial dysfunction plays a key role in the pathogenesis of several human diseases, including chronic lung diseases. Schuliga *et al.*[32] investigated the role of the DNA-sensing guanine monophosphate-adenine monophosphate (GMP-AMP) synthase (cGAS) in IPF. AECs from IPF lung tissue displayed higher baseline senescence than control AECs, as assessed by increased nuclear histone 2AXγ phosphorylation, p21 mRNA and expression of SASP cytokines, whereas inhibition of cGAS diminished IPF-AEC senescence and attenuated induction of control AEC senescence following etoposide-induced DNA damage, identifying cGAS as a potential therapeutic target in IPF. Phosphoglycerate mutase family member 5 (PGAM5), a mitochondrial protein with a crucial role in mitochondrial dynamics and programmed cell death, may also contribute to the pathogenesis of pulmonary fibrosis. Indeed, Ganzleben *et al.*[33] have shown that PGAM5 alters mitochondrial integrity both functionally and structurally whereas PGAM5 deficiency improves mitochondrial homeostasis and attenuates lung fibrosis in mice.

Peroxisome proliferator activated receptor gamma co-activator 1-alpha (PPARGC1A, encoding PGC1α) is a master regulator of mitochondrial biogenesis. In normal human lung fibroblasts, PGC1α knockdown reduces mitochondrial mass and function while enhancing senescence-related gene expression and profibrotic signalling whereas re-expression of PGC1α in IPF fibroblasts ameliorates all of these pathological cellular functions [34]. This data identifies PGC1α as an important regulator of the fibroblast pathological state in IPF. Recently, Chung *et al.*[35] have demonstrated that the absence of the mitochondrial fusion proteins mitofusin1 (MFN1) and 2 (MFN2) in murine AEC2 cells leads to spontaneous lung fibrosis. In addition, they found that MFN1 and MFN2 are important regulator of the synthesis of phospholipids and cholesterol in AEC2 cells, and hypothesized that mitochondrial fusion and lipid metabolism are tightly linked to regulate AEC2 cell injury and fibrotic lung remodeling.

Mitochondrial dysfunction and endoplasmic reticulum (ER) stress are generally regarded as separate mechanisms contributing to the pathogenesis of IPF. Jiang *et al.*[36] have shown that activating transcription factor 4 (ATF4) is a key regulator of mitochondrial unfolded protein response (UPRmt) both in experimental pulmonary fibrosis and in IPF, and that this signalling cascade is induced by ER stress, indicating a novel mechanism by which ER stress may contribute to the pathogenesis of IPF.

### Chronic obstructive pulmonary disease

Tobacco-induced mitochondrial dysfunction has been implicated in the pathogenesis of COPD [37]. Specifically, CS extract (CSE) induces failure to scavenge mitochondrial reactive oxygen species (mtROS), which are important for mitochondrial function and integrity, leading to decreased ATP levels and mitochondrial membrane potential (MMP), and impaired mitophagy [22,38,39].

Haji and co-workers examined mitochondrial function in mitochondria isolated from bronchial biopsies and quadriceps muscle from patients with COPD and smokers without COPD and found increased mtROS levels in both study groups [40]. However, decreased MMP and superoxide dismutase 2 (SOD2) levels were observed only in mitochondria isolated from bronchial biopsies from COPD GOLD 2 patients, suggesting that mitochondrial events in the quadriceps muscle may be dissociated from those in the airways. Mitochondrial dysfunction and increased mtROS levels have also been demonstrated in airway smooth muscle cells from patients with COPD compared with nonsmokers [41]. Of note, smokers without COPD displayed intermediate mitochondrial dysfunction, suggesting that these alterations are not due solely to cigarette smoke but also to COPD itself.

Unlike other organelles, mitochondria have their own maternally inherited DNA (mtDNA) [42], and mtDNA replication is important for mitochondrial growth and division [43]. Mitochondrial dysfunction is often associated with the leakage of mtDNA, which may be detected extracellularly in various bodily fluids, such as plasma and urine [44,45]. In the SPIROMICS cohort, plasma levels of mtDNA levels did not differ between nonsmokers and smokers without COPD, but were higher in mild or moderate COPD subjects. Moreover, plasma mtDNA levels were lower in severe COPD subjects compared to mild or moderate COPD, suggesting that mitochondrial dysfunction may induce disease worsening [46^▪^]. In another study from the SPIROMICS cohort, Zhang *et al.*[47] found that urinary mtDNA was associated with worse distance at 6-min walking test, St. George Respiratory Questionnaire (SGRQ), and COPD assessment test (CAT) score.

This data suggests that mitochondrial dysfunction may represent a potential therapeutic target in COPD. Mitochondria-targeted antioxidants such as mitoQ, mito-TEMPO, pyrroloquinoline quinone, and SkQ1 are more effective than conventional antioxidants in preventing (or reversing) age-associated mechanisms in rats [48]. In addition, in human airway epithelial cells, mito-TEMPO inhibits mitochondrial dysfunction and mtROS release following CSE [49] whereas mitoQ reduces neutrophilic inflammation, airway hyperresponsiveness and lung inflammatory mediators in a mouse model of chronic oxidative stress [41].

## AUTOPHAGY

### Idiopathic pulmonary fibrosis

Autophagy is a cellular mechanism for homeostasis and response to stress that has recently emerged as a potential contributor to the initiation and progression of several chronic lung diseases, including IPF. Specifically, insufficient autophagy may contribute to disease pathogenesis by promoting epithelial-to-mesenchymal transition (EMT) and aberrant epithelial-fibroblast crosstalk [50]. In addition, reduced autophagy results in excessive generation of ROS, leading to oxidative stress and cell damage. Tian *et al.*[51^▪^] have recently shown that the expression of Leucine Rich Repeat Kinase 2 (LRRK2), a protein with an important role in lung homeostasis, is significantly reduced in mammalian fibrotic lungs. LRRK2 deficiency results in dysfunctional AT2 cells, including impaired autophagy and accelerated cellular senescence, thus identifying a potential therapeutic target in pulmonary fibrosis. Tsoy *et al.*[52^▪▪^] investigated the role of CD148/PTRJ (receptor-like protein tyrosine phosphatase η), a tyrosine phosphatase with antifibrotic activity in human pulmonary fibrosis. They showed that CD148 expression was downregulated in IPF lungs and fibroblasts, and that CD148-deficient fibroblasts exhibited hyperactivated PI3K/Akt/mTOR signalling and reduced autophagy, suggesting that targeting the CD148 phosphatase may represent a therapeutic strategy in IPF. Interleukin (IL)-37 enhances beclin-1-dependent autophagy and autophagy modulators in IPF fibroblasts, and significantly decreases collagen deposition in bleomycin-exposed mouse lungs, lending further support to the efficacy of autophagy augmentation strategies in pulmonary fibrosis [53].

### Chronic obstructive pulmonary disease

The role of aberrant autophagy in the pathogenesis of COPD remains to be established. In the lung epithelium, activation of autophagy in response to CS has been associated with airway inflammation and mucus hypersecretion [54,55]. In this regard, Song *et al.*[56] recently reported that ghrelin, a peptide hormone that regulates food intake, body weight and glucose homeostasis, inhibits autophagy and inflammation induced by particle matter (PM) and/or CS exposure in bronchial epithelial cells *in vitro* and *in vivo*. However, other studies have shown that loss of autophagy enhances epithelial cell senescence, mtROS production and accumulation of ubiquitinated proteins, suggesting a protective role for autophagy in COPD pathogenesis [57–59].

Multiple types of organelle-specific autophagy have been described, including mitophagy, pexophagy, reticulophagy, ribophagy, lysophagy, and nucleophagyan [60]. Several studies have shown that CSE may lead to impaired mitophagy, with consequent accumulation of damaged mitochondria [58,61,62]. The PINK1 (PTEN-induced putative kinase 1)−PARK2 pathway has also been proposed as a crucial mechanism for mitophagic degradation, and inhibition of PINK1−PARK2 pathway-mediated mitophagy results in elevated ROS generation and inflammasome activation in small airway epithelial cells from COPD patients [58]. Dysregulated autophagy may occur both in early stages of COPD, wherein CSE induces microtubule-associated protein-1 light chain-3B (LCB3) expression, thus enhancing autophagy [62], and in advanced stages, with diminished mitophagy accelerating cellular senescence and damage [58].

## CONCLUSIONS

**FIGURE 1:**
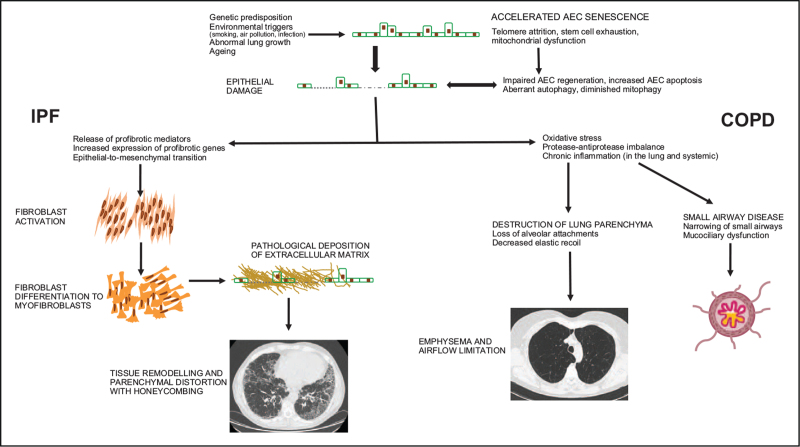
Proposed models of how cellular senescence may contribute to the pathogenesis of IPF and COPD. Abbreviations: AEC, alveolar epithelial cell; COPD, chronic obstructive pulmonary disease; IPF, idiopathic pulmonary fibrosis.

IPF and COPD are two progressive and irreversible respiratory diseases. Several ageing mechanisms are dysregulated in the lungs in both diseases, resulting in pathological tissue repair, rather than wound resolution, following injury (Fig. 1). However, the prevalence, pathology and clinical behaviour of IPF and COPD differ markedly. This is likely to result from substantially different genetic background and epigenetic changes between the two diseases leading to differences in the target cell types and molecular responses to environmental triggers, including smoking. The mechanisms that contribute to the age-associated predilection of IPF and COPD remain elusive. As our understanding of the cellular and molecular mechanisms that ultimately converge to drive the abnormal remodelling of the aged lung improves, opportunities will emerge to identify novel targets and therapeutic strategies for IPF (and possibly COPD).

## Acknowledgements

*None.*

### Financial support and sponsorship

*None.*

### Conflicts of interest

*There are no conflicts of interest*.

## REFERENCES AND RECOMMENDED READING

Papers of particular interest, published within the annual period of review, have been highlighted as:

▪ of special interest
▪▪ of outstanding interest
